# Exploration and retrieval of whole-metagenome sequencing samples

**DOI:** 10.1093/bioinformatics/btu340

**Published:** 2014-05-19

**Authors:** Sohan Seth, Niko Välimäki, Samuel Kaski, Antti Honkela

**Affiliations:** ^1^Helsinki Institute for Information Technology HIIT, Department of Information and Computer Science, Aalto University, Espoo, Finland, ^2^Genome-Scale Biology Program and Department of Medical Genetics, University of Helsinki, Helsinki, Finland, and ^3^Helsinki Institute for Information Technology HIIT, Department of Computer Science, University of Helsinki, Helsinki, Finland

## Abstract

**Motivation:** Over the recent years, the field of whole-metagenome shotgun sequencing has witnessed significant growth owing to the high-throughput sequencing technologies that allow sequencing genomic samples cheaper, faster and with better coverage than before. This technical advancement has initiated the trend of sequencing multiple samples in different conditions or environments to explore the similarities and dissimilarities of the microbial communities. Examples include the human microbiome project and various studies of the human intestinal tract. With the availability of ever larger databases of such measurements, finding samples similar to a given query sample is becoming a central operation.

**Results:** In this article, we develop a content-based exploration and retrieval method for whole-metagenome sequencing samples. We apply a distributed string mining framework to efficiently extract all informative sequence *k*-mers from a pool of metagenomic samples and use them to measure the dissimilarity between two samples. We evaluate the performance of the proposed approach on two human gut metagenome datasets as well as human microbiome project metagenomic samples. We observe significant enrichment for diseased gut samples in results of queries with another diseased sample and high accuracy in discriminating between different body sites even though the method is unsupervised.

**Availability and implementation:** A software implementation of the DSM framework is available at https://github.com/HIITMetagenomics/dsm-framework.

**Contact:** sohan.seth@hiit.fi or antti.honkela@hiit.fi

**Supplementary information:** Supplementary data are available at *Bioinformatics* online.

## 1 INTRODUCTION

Metagenomics is the study of microbial communities in their natural habitat using genomics techniques (Tyson *et al.*, 2004). It is undergoing a boom owing to the proliferation of high-throughput sequencing technologies. Many studies focus at targeted sequencing of specific marker genes such as the 16S rRNA gene in bacteria, but recently there has been a growing interest in whole-metagenome sequencing (e.g. Human Microbiome Project Consortium, 2012; Qin *et al.*, 2010). Although targeted studies provide data for phylogenetic profiling at a lower cost, whole metagenomes provide much more information, for example, about the collective metabolism (Greenblum *et al.*, 2012) and the population genetics of the community (Schloissnig *et al.*, 2013). Recent studies have also found associations between features of whole human gut metagenomes and type II diabetes (Qin *et al.*, 2012). New data are accumulating rapidly, with a popular web-based MG-RAST server (Meyer *et al.*, 2008) listing almost 3000 public whole metagenomes.

Analyzing whole-metagenome shotgun (WMS) sequencing data is very challenging. The original sample typically contains genetic material from hundreds to thousands of bacterial species of different abundances (Li *et al.*, 2012), most of which have not been fully sequenced previously. After sequencing, we obtain a huge collection of short sequence reads whose species of origin is unknown. Although significant progress has been made, analysis relying on either the limited previously annotated genomes, or assembling the reads into novel more complete genomes, remains difficult and inefficient, and potentially susceptible to annotation biases.

In this article, we introduce an efficient purely data-driven feature extraction and selection method as well as similarity measures for WMS sequencing datasets, and apply them in retrieval of similar datasets. Such content-based retrieval is an extremely powerful tool for exploration of the data and generating hypotheses of disease associations, as previously demonstrated with gene expression data (Caldas *et al.*, 2009, 2012). Retrieval from existing databases makes it possible to automatically explore a much greater variety of hypotheses than relying solely on the more common specifically designed focused studies.

Content-based similarity measures and retrieval of similar metagenomic datasets have been suggested previously (Jiang *et al.*, 2012; Liu *et al.*, 2011; Mitra *et al.*, 2009; Su *et al.*, 2012), based on quantifying abundances over a relatively small number of predetermined features requiring existing annotation. Up to some thousands of known taxa, genes or metabolic pathways have been used. We introduce similarity measures that are based solely on raw sequencing reads, and hence, unbiased and insensitive to the quality of the existing annotation. A similar measure has been previously suggested by Maillet *et al.* (2012), but only for pairwise comparisons using a method that is computationally too expensive to scale to even modestly large datasets. Furthermore, instead of considering all sequences of particular length, also known as *k*-mers, as has been done earlier for other tasks and by Maillet *et al.* (2012), we employ an efficient distributed string mining (DSM) algorithm to find informative subsequences that can be of *any* length.

To deal with the large number of features, some feature selection is necessary. Previous approaches for detecting relevant features in metagenomic data have been based on direct comparison of two classes of samples. Again, most of these methods work on up to some thousands of features (Parks and Beiko, 2010; Segata *et al.*, 2011; White *et al.*, 2009), with the notable exception of one study (Qin *et al.*, 2012) where quantification and association testing was done for >4.3 million predefined genes. Without feature selection, one can use short *k*-mers (Baran and Halperin, 2012) or limit to a set of *k*-mers that are likely to be informative, such as *k*-mers associated with well-characterised protein families (Edwards *et al.*, 2012). Although there are no previous examples of unsupervised feature selection for metagenomics, it is a common practice in information retrieval with text documents (Yang and Pedersen, 1997); a particularly relevant method assesses the entropy of the distribution of documents in which a specific term occurs (Largeron *et al.*, 2011).

We evaluate the performance of the proposed unsupervised, unconstrained retrieval method on synthetic data, as well as metagenomic samples from human body sites (Human Microbiome Project Consortium, 2012; Qin *et al.*, 2010, 2012). To evaluate the performance of the retrieval engine, we use external validation based on a ground truth similarity between two samples. To simplify this process, we consider a binary similarity, which is crude but easily accessible. The human gut samples in (Qin *et al.*, 2010, 2012) come from studies exploring the change in bacterial species composition between healthy persons and either inflammatory bowel disease (IBD) or type II diabetes. We utilize disease state to construct a binary ground truth. Thus, we study if, given the metagenomic sample of a person with a disease, the retrieval finds metagenomic samples related by having the same disease. In the body site data (Human Microbiome Project Consortium, 2012), we use the body sites as ground truth to investigate whether it is possible to identify the bacterial communities at different body sites in an unsupervised setting without the need of reference genomes. It should be noted that especially for the gut data, two samples may be related in other ways too. The external validation with one simple ground truth nonetheless provides an objective platform for comparing different methods. Given that the method is unsupervised and hence completely oblivious of the disease labels, if such retrieval is successful, it is a promising starting point for developing methods for leveraging data from earlier patients in early detection of disease and personalized medicine.

## 2 APPROACH

**Fig. 1.**
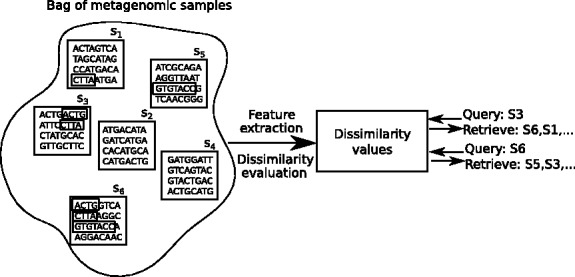
Given a set of metagenomic samples, our objective is to be able to retrieve relevant samples to a query sample. For this, we need to extract relevant features and evaluate a pairwise similarity (or dissimilarity) measure. The samples are then ranked in the order of increasing dissimilarity from the query

Our objective is to extract and select suitable features for representing WMS sequencing samples and to form a pairwise dissimilarity measure for a collection of such samples. Given this dissimilarity, one can query with a sample and retrieve other samples that are similar to it (Fig. 1). The measure needs to be reasonably rapidly computable, yet captures relevant differences between the samples, and does all this with as little prior biological knowledge and annotations as possible, as detailed quantitative prior knowledge is typically not yet available for metagenomics.

Evaluating dissimilarity requires representing the metagenomic sample in a suitable feature space. A standard choice for representing objects over strings is to estimate the *k*-mer frequency values, where a *k*-mer here is a string of *k* letters from the DNA alphabet {A,C,T,G}. Therefore, there are 4*^k^* possible *k*-mers for any given *k*. It is a standard practice to set *k* to a specific value, typically a small value to keep the estimation problem tractable both computationally and statistically. A larger *k* would give better discriminability but not without bounds, as for finite dataset sizes there simply are not enough data to estimate long *k*-mers. We argue that instead of setting *k* to a particular value, it is more effective to estimate all possible *k*-mers for all possible *k* which the data supports. This makes the problem more challenging, as the number of such observed different *k*-mers for large *k* becomes very large, and they become more susceptible to sequencing errors. Focusing on *k*-mers appearing more than once in a sample helps significantly because it is relatively rare to have the exactly same sequencing errors in two independent reads.

To make the method computationally efficient, we treat each *k*-mer as an independent feature. We compute a Bayesian estimate of their relative frequencies across samples. The employed prior helps in suppressing noise caused by small observed read counts. In the *filtering* step, the abundance distribution of each *k*-mer over samples is used to judge informativeness of the *k*-mer for retrieval; a *k*-mer with constant abundance does not have discriminative power and, in the other extreme, a *k*-mer which is present in only one sample cannot generalize over samples. We show that the filtering step significantly improves the retrieval performance with most datasets and distance measures. Finally, we compute the dissimilarity between two samples across the features as a weighted average of distances between relative frequencies of individual *k*-mers. Treating each *k*-mer as an independent feature allows us to execute these steps fast and on the fly without storing the intermediate results. Such simplified distance measures are necessary to guarantee scalability given the extremely high dimensionality of the *k*-mer features.

**Fig. 2.**
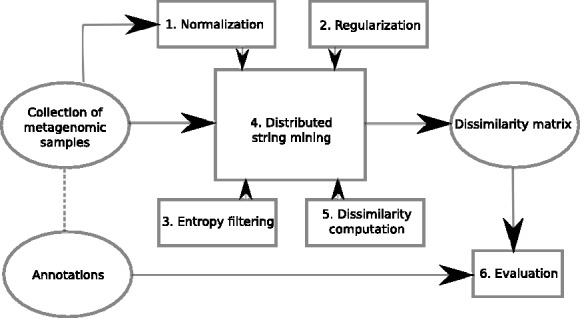
Processing steps of our method. Given a collection of metagenomic samples, we use the collection as an input to the DSM method (4). For the method, we estimate the frequency of each *k*-mer (1, 2), evaluate if the *k*-mer is informative or not (3), and compute the needed dissimilarities (5). Finally, in this article we evaluate the performance considering the existing annotations as ground truth; annotations are not needed for the retrieval in general

To summarize, we introduce methods to (i) estimate the frequencies of a large number of *k*-mers over multiple samples, (ii) decide if a *k*-mer is informative or uninformative in the context of a retrieval task, (iii) compute a distance metric using the filtered *k*-mer frequencies, and (iv) execute these steps fast without explicitly storing the frequency values. Figure 2 summarizes the method.

## 3 METHODS

### 3.1 Estimating *k*-mer frequencies: normalization, regularization and filtering

To perform the feature selection or filtering, we first compute Bayesian estimates of the relative frequencies p(s|w) of each *k*-mer *w* over samples s∈S using observed frequencies $\hat{f}(s,w)$ of the *k*-mers. These are distributions over samples for each *k*-mer that are computed independently for each *k*-mer for reasons of computational efficiency.

Even if the relative abundance of a *k*-mer is the same in every sample, the observed frequencies may differ because of different sequencing depth or coverage in different samples. To tackle this issue, we employ normalization: we normalize the frequency $\hat{f}(s,w)$ by a sample-specific constant σ(*s*), which is proportional to the total number of base pairs in a sample, and σ(s)=1 for the largest sample in the collection in terms of total base pair count, obtaining

(1) $$f(s,w)=\hat{f}(s,w)/\sigma (s).$$

The σ(*s*) can be interpreted probabilistically as the probability of observing a sequence in the actual sample, assuming every sample had the same number of base pairs to start with, but some have been lost in the processing.

To estimate the relative frequencies, we place a conjugate symmetric Dirichlet prior on the parameters of the multinomial distribution over the observed counts. The common choice of uniform prior distribution corresponds to a Dirichlet distribution with all parameters equal to 1. This yields a posterior mean estimate of the relative frequency values as

(2) $$p(s|w)=\frac{f(s,w)+1}{\sum_{s\prime \in S}\left[f(s\prime ,w)+1\right]}.$$

The Dirichlet prior with all parameters equal to 1 is ubiquitous in document retrieval. It is particularly suitable for metagenomics owing to the following observations: First, the DSM algorithm (described below) trades off low *k*-mer counts for speed and ignores any *k*-mers that are present only once in a sample. The pseudocount from the prior makes up for this missing count. Second, adding pseudocounts assists in playing down the significance of rare *k*-mers that may appear due to sequencing errors in the filtering step without affecting other *k*-mers too much.

Finally, given the massive number of potential *k*-mers, it is crucially important to improve signal-to-noise ratio by focusing on the informative ones. For the unsupervised tasks of comparing the samples, obviously only *k*-mers that distinguish between the samples are informative. As a concrete example, consider a *k*-mer that is present in all samples with a similar abundance. It certainly does not give information useful for comparing samples. In the other extreme, if a *k*-mer is present in one specific sample, but not in any other, it is potentially a spurious *k*-mer due to sequencing error, and in any case does not help in comparing samples either. On the other hand, if a *k*-mer is present in some samples, but not all, then it gives information that those samples are similar in a specific sense. Informativeness in this sense can be measured by the entropy *H* of the distribution of the *k*-mer over the samples: we filter the *k*-mers based on the conditional entropies

(3) $$H(S|w)=-\frac{1}{\log(|S|)}\sum_{s\in S}p(s|w)\log p(s|w);$$

a *k*-mer is taken into account in distance computation only if the normalized entropy is lower than a certain threshold *e*. By design 0≤H≤1. Notice that in standard *information theory* terminology higher entropy implies higher information. However, in our context an informative *k*-mer has low entropy. Also, due to the Bayesian estimation, a spurious *k*-mer having only small counts will have large conditional entropy and will be filtered out.

The optimal value of threshold *e* varies with datasets. It can be ‘optimized’ in a supervised manner by utilizing a *training set* where we have labeled samples. In the absence of a labeled set, we suggest taking the ‘average’ of distance metrics computed over the potential thresholds as the final metric. We refer to the final metrics in the two cases as *optimized metric* and *average metric*. In our experimental set-up, we randomly make a 50–50 split of a given dataset in training $S_{\text{tr}}$ and testing $S_{\text{te}}$ sets: $S_{\text{tr}}\cap S_{\text{te}}=\emptyset$ and $S_{\text{tr}}\cup S_{\text{te}}=S$. We use $S_{\text{tr}}$ to optimize the entropy threshold: we query with samples in $S_{\text{tr}}$ and retrieve relevant samples within the same set to observe which entropy threshold results in the best retrieval result (see Section 3.4 for details). While comparing the performance of two methods we always present the evaluation over $S_{\text{te}}$: we query with samples within $S_{\text{te}}$, and we retrieve relevant samples from *S* (not just $S_{\text{te}}$). Notice that the training set can also be used to judge the importance of individual features.

### 3.2 Algorithms to extract informative *k*-mers

Our main computational challenge is to extract all informative *k*-mers from large-scale datasets in feasible time and space. Recall that the filtering step relies on knowledge over multiple samples to decide if the respective *k*-mer is informative for the retrieval task or not. Because the typical collections of WMS samples are huge in size, we cannot assume that even the plain input fits into the main memory of any single machine. To process these large-scale datasets, the computation needs to be done either using external memory (i.e. disk) or in a distributed manner (i.e. a computer cluster). We review two approaches: *k-mer counting* (Marais and Kingsford, 2011; Rizk *et al.*, 2013) and DSM (Välimäki and Puglisi, 2012). The first one is a standard approach in the literature for fixed *k*, but has several limitations when applied in our context of multiple samples and large-scale data. We show that the latter approach is more flexible in this context and can also be generalized to extract informative *k*-mers over all values of *k* simultaneously.

Jellyfish (Marais and Kingsford, 2011) and DSK (Rizk *et al.*, 2013) are examples of recent algorithmic improvements in *k*-mer counting. Both tools use hash tables to compute the *k*-mer distribution for a given (fixed) *k*. In both tools, space efficiency is achieved by keeping most of the hash table on disk. The main drawback with these disk-based approaches is that they are aimed at counting *k*-mers in a single sample and extending them over to multiple samples is non-trivial. For example, Jellyfish could, in principle, be extended to count *k*-mers over multiple samples: the authors give a roughly linear time algorithm to merge two or more hash tables. However, the intermediate *k*-mer counts would need to be stored on disk, which requires significant amount of additional space, and the merge phase is not parallelized (Marais and Kingsford, 2011, User manual, Section Bugs).

The decision whether a particular *k*-mer is informative or not is made by looking at its frequency over all the given WMS samples. We tackle this problem by a DSM framework (Välimäki and Puglisi, 2012) that can handle multi-sample inputs by utilizing a computer cluster. The main advantages of this framework are that (i) load balancing divides the data and computation over multiple cluster nodes, (ii) intermediate *k*-mer counts are not stored explicitly and (iii) there is no additional disk I/O strain, except reading through the input once. These advantages allow terabyte-scale data analysis on a cluster having limited main memory per node, but a sufficient number of nodes available to facilitate the computation in distributed manner. We extend the DSM framework to be compatible with our definition of informative *k*-mers (see the above subsection). It allows us to extract the informative *k*-mers either for a fixed *k* or over all values of *k* in feasible time.

**Fig. 3.**
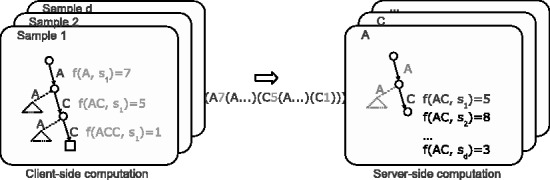
Technical overview of our DSM framework consisting of client (left) and server (right) processes. The client-side processes are responsible for computing the substring frequencies within each sample $s_{1},s_{2},\ldots s_{d}$ separately. Substrings and their frequencies are found using a depth-first-traversal over a (compressed) suffix tree. Frequency information is transmitted over to the server-side by streaming it as a balanced-parenthesis representation of a sorted trie. For example, the trie on the left results as the parenthesis representation given in the middle. The server reads the client-streams and merges the (already sorted) tries in recursive manner: at each node, the server computes the entropy based on the received values and updates the affected pairwise distances. Load balancing on the server-side is achieved by hashing the prefix of the substring so that each server corresponds to a certain range of hash values

The DSM framework is based on a client-server model. The clients have one-to-one correspondence to the given samples, each client being responsible for computing the frequencies within the designated sample. The client-side computation relies heavily on *suffix sorting* techniques and on space-efficient data structures for strings (Välimäki and Puglisi, 2012): the input data are first preprocessed into a compressed representation, which replaces the input data and acts as an efficient search structure. The server-side computation is more straightforward: the server simply merges the (sorted) input from the clients, computes the entropies and updates the distance matrices. Figure 3 gives a toy example of the client–server interaction. Two crucial observations are needed to keep the whole computation and transmission costs feasible. First, the informative *k*-mers can be seen as a subset of *left–right-branching* substrings, i.e. substrings whose instances have differentiating continuation on both left and right. More formally: substring *w* of string *T*[1,*n*] is called *right-branching* if there exists two symbols *a* and *b* such that a≠b and both *wa* and *wb* are substrings of *T*. Similarly, a substring *w* is *left-branching* if *aw* and bw,a≠b, are substrings of *T*. If a substring is both left-branching and right-branching, we say it is *left–right-branching*. Second, for any string of length *n*, there are at most *O*(*n*) left–right-branching substrings, and the total length of all such substrings is bounded by O(nlog⁡n) (Kärkkäinen *et al.*, 2009, Theorem 1).

The first observation allows us to reduce the client-side computation to a smaller set of substrings: it is easy to see that if *k*-mer *w*, having frequency f′(s,w)≥2, is non-branching, then there exists a substring w′ of length k′>k that is left–right-branching and has exactly the same frequency, i.e. f′(s,w)=f′(s,w′). It follows that the frequency of non-branching *k*-mers can be deduced from the branching k′-mers, and the left–right-branching substrings contain all the necessary information for us to detect informative *k*-mers. The second observation guarantees a feasible transmission cost between clients and servers: the upper bound for the concatenation of all left–right-branching substrings also acts as an upper bound for both the server-side running time and the amount of communication needed. The drawback of restricting to left–right-branching substrings is that the informative *k*-mers that we are able to detect have to appear at least twice in a sample, although this limit may be useful in pruning spurious *k*-mers introduced by sequencing errors. More detailed explanation and analysis of the DSM framework is given in Välimäki and Puglisi (2012). A software implementation of the DSM framework is available at https://github.com/HIITMetagenomics/dsm-framework.

### 3.3 Dissimilarity metrics

Having extracted the informative *k*-mers, we use them to compute the dissimilarity between two metagenomic samples. We consider three dissimilarity metrics that can be computed easily over a large number of *k*-mers in sequential manner, i.e. one *k*-mer at a time, and without storing all the *k*-mer frequencies explicitly. To utilize the natural variance structure of the *k*-mers—some are more abundant than others—we weight the relative frequencies of each *k*-mer by their respective total counts, i.e. we utilize the absolute frequencies f(s,w) as defined in (1).

We mainly use the simple Jaccard distance that does not consider abundances at all, only whether a *k*-mer occurs or not. Given two sets *s*_1_ and *s*_2_ of *k*-mers detected as present in two different samples, Jaccard distance measures how many elements are shared between these two sets. Mathematically, it is defined as

$$D_{\text{count}}(s_{1},s_{2})=1-\frac{\left|s_{1}\cap s_{2}\right|}{\left|s_{1}\cup s_{2}\right|}.$$

Despite its simplicity, we observe that Jaccard distance performs well; a potential reason is its robustness to measurement noise and effectiveness when two metagenomic samples differ in terms of presence and absence of certain species or functionalities. We assume a *k*-mer is present in a sample if its frequency is >2.

We also experiment with two metrics that use the abundance information:

I. *Variance-stabilized Euclidean distance:* An obvious distance measure between two metagenomic samples *s*_1_ and *s*_2_ is the Euclidean distance between their respective *k*-mer frequencies. We consider the distance metric

$$D_{\text{sqrt}}(s_{1},s_{2})=\sum_{w}{\left(\sqrt{f(w,s_{1})}-\sqrt{f(w,s_{2})}\right)}^{2}$$

which can be computed sequentially as new informative *k*-mers are extracted. The square root transformation is the *variance stabilizing transformation* for Poisson distribution—a popular model for quantitative sequencing data.

II. *Log transformed Euclidean distance:* We also consider the same metric but with log transformation, which is a popular approach in document retrieval, i.e.

$$D_{\text{log}}\left(s_{1},s_{2}\right)=\sum_{w}{\left(\text{log}\left(1+f\left(w,s_{1}\right)\right)-\log\left(1+f\left(w,s_{2}\right)\right)\right)}^{2}.$$

The motivation for using the log transformation is that it decreases sensitivity to high frequency counts: some *k*-mers are present in high abundance in almost every genome, for instance *k*-mers from the marker gene, and the log transformation reduces their effect in the metric.

### 3.4 Evaluation metric

We evaluate the performance of the dissimilarity metric in terms of its performance in the task of retrieving relevant samples given a query metagenomics sample. The ground truth for relevance is either the disease class (disease versus not) or the known body site: samples from the same class are considered relevant.

For measuring retrieval performance, we use an evaluation metric which is popular in document retrieval, the mean average precision (MAP) (Smucker *et al.*, 2007). Given a query *q*, the retrieval method ranks the samples in an increasing order of their dissimilarities from *q*. Given one has retrieved the top (closest) n∈{1,…,N} samples the precision @ *n* is defined as

$$\text{Precision}(n;q)=\frac{\text{number of relevant samples in}\;n\;\text{retrieved samples}}{n},$$

and MAP defined using *average precision* as,

$$\text{MAP=}\frac{1}{\left|Q\right|}\sum_{q\varepsilon Q}\text{AveP}\left(q\right),\text{AveP}\left(q\right)=\frac{1}{m_{q}}\sum_{n\in R_{q}}\text{Precision}\left(n;q\right).$$

Here, *Q* is the set of all queries, *m_q_* is the number of relevant samples to query *q* and *R_q_* is the set of locations in the ranked list where a relevant sample appears. It is straightforward that a higher MAP implies better performance. To judge if two MAP values are significantly different or not, we employ the randomization test described in Smucker et al. (2007): for each query, this test randomly reassigns the AvePs achieved by two methods to one another, and computes the difference between the resulting MAP for multiple such reassignments to get a distribution, against which the true MAP value is tested in terms of *P*-value. In case two samples share the same dissimilarity from a query sample, we employ the modification suggested in McSherry and Najork (2008) to break ties. When computing the mean, we follow a leave-one-out cross-validation type approach using each sample as a query, and retrieving from the rest of the collection. For simulated data and human gut samples, we only query with the positive samples in the testing set $q\in S_{\text{te}}$, whereas for body site samples we query with each sample in the testing set. For both cases we retrieve from the entire set S∖{q}. Although choosing the entropy threshold in a supervised setting, we query from $q\in S_{\text{tr}}$ and retrieve from $S_{\text{tr}}\setminus \{q\}$.

### 3.5 Synthetic data generation

To test the method, we simulated four datasets containing samples from separate classes, with the interpretation that samples from the same class are relevant. In all the datasets we have two classes: both classes of samples have the same species composition but different relative abundances. We used MetaSim (Richter *et al.*, 2008) to generate Illumina reads of length 80 using the error configuration file provided by the developers. Each dataset contains 200 samples: 98 of them belong to the *positive* class and the rest belong to the *negative* class. For each dataset, we used the same 100 species from the following genera: acetobacter, acetobacterium, acidiphilium, acidithiobacillus, acinetobacter, bacillus, bacteroides, bifidobacterium, chlamydia, chlamydophila, clostridium, escherichia, haloarcula, halobacterium, lactobacillus, pasteurella, salmonella, staphylococcus and streptococcus. The abundance profiles were generated from two Dirichlet distributions; one for positive and the other for negative class. The parameters of the Dirichlet distributions were shared between two classes: for half of the species (randomly chosen) the same parameters were used for both classes and for the other half of the species the parameters were randomly permuted. For example, given five species the assigned parameters could be: (0.3, 0.2, 0.6, 0.1 and 0.9) and (0.9, 0.2, 0.3, 0.1 and 0.6) where the parameters for the second and fourth species are the same, but for the other species they were permuted. The exact species and corresponding parameter values can be downloaded from https://github.com/HIITMetagenomics. The resulting datasets are—HIGH-C, relatively easy data with high coverage (10*e*6 reads/sample); LOW-C, relatively difficult data with low coverage (2*e*6 reads/sample); MIXED-C, mixed data with half the samples from HIGH-C and the rest from LOW-C to simulate varying sequencing depth; and HIGH-VAR, relatively difficult data with same coverage as HIGH-C but additional noise in the class distributions to simulate more overlap between classes. To elaborate, the relative abundance of species is $p_{\text{HIGH-VAR}}=0.5p_{\text{HIGH}}+0.5noise$ where *noise* is generated from a symmetric Dirichlet distribution with all parameters equal to 1.

## 4 RESULTS

We evaluated the retrieval performance on three human metagenomics datasets:

1. MetaHIT (Qin *et al.*, 2010), 124 metagenomic samples from 99 healthy people and 25 patients with IBD syndrome. Each sample has on average 65 ± 21 million reads. Our goal was to retrieve IBD-positive patients.
2. T2D Phase II (Qin *et al.*, 2012), 199 metagenomic samples from 100 healthy people and 99 patients with type II diabetes. Each sample has on average 47 ± 11 million reads. Our goal was to retrieve patients with diabetes. We chose to explore the phase II data instead of the phase I data, as the former has higher coverage; about 40% more reads than the latter.
3. HMP (Human Microbiome Project Consortium, 2012), 435 metagenomic samples from 10 different body sites (see Supplementary Table S1). Of 690 samples that passed the QC assessment (http://www.hmpdacc.org/HMASM/), we discarded 255 samples that had <1% of the number of reads of the largest sample.

To recapitulate, for MetaHIT and T2D-P2, our goal is to observe if given a positive sample, e.g. from a patient with a particular disease, one can retrieve relevant samples, i.e. with similar disease; whereas for HMP, our goal is to observe if given a sample from a particular body site, one can retrieve relevant samples, i.e. samples from the same body site. For all data, we applied a quality threshold of 30 and ignored any base pairs with quality less than the threshold. Table 1 gives an overview of the computational resources required for each dataset. Additionally, number of *k*-mers used by different methods for each dataset are available in the Supplementary Fig. S1.

**Table 1.** Computational resources required by the DSM on different datasets

	HIGH-C	MetaHIT	T2D-P2	HMP
Input size (GB)	149	536	786	3353
Samples	200	124	199	435
Preproc. (h)	0.4	3.6	10	65
Total memory (GB)	117	209	610	2885
All *k*				
Wall clock (h)	4.9	2.0	8.0	53
CPU time (h)	149	187	1137	20 000
*k* = 21				
Wall clock (h)	1.8	0.4	2.8	12
CPU time (h)	10	74	279	4000

*Note:* We report wall-clock times and total CPU times for both fixed *k* = 21 and over all *k*. Preprocessing is done only once, separately from the actual computation. Total memory is the memory requirement over all computation nodes. Experiments were run on a cluster of Dell PowerEdge M610 nodes having 32 GB of RAM and 16 cores. Simulated data and MetaHIT were run using up to eight nodes. T2D-P2 was run using 32 nodes allowing more parallelization at the server-side. HMP was run on a cluster of 20 nodes with 2 × 10-core Xeon CPUs and 256 GB RAM.

**Fig. 4.**
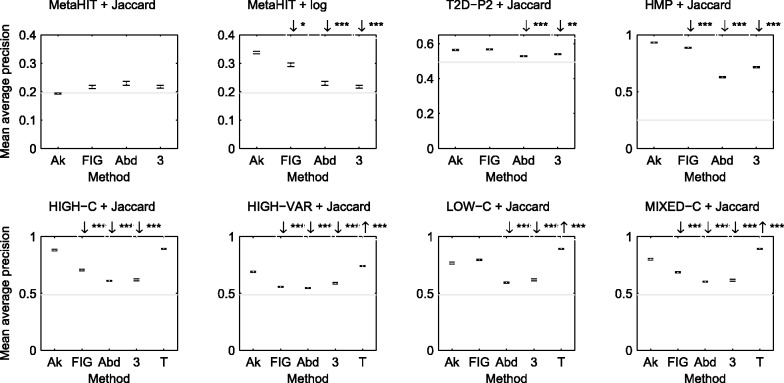
Retrieval performance comparison of the proposed approach using all *k*-mers (‘Ak’) against the following base measures: (1) ‘FIG’: retrieval performance using known protein family, (2) ‘Abd’: Bray–Curtis dissimilarity between relative estimated abundance, (3) ‘3’: $d_{S}^{2}$ distance between relative abundance of 3-mers. ‘Ak’ uses the ‘optimized metric’ over 101 equally spaced threshold values between 0 and 1. Each error bar shows the MAP value along with the standard error. The grey horizontal line shows retrieval by chance: MAP computed over zero similarity metric. An arrow (if present) over a method indicates whether the performance of the corresponding method is significantly better (↑) or worse (↓) than ‘Ak’: The stars denote significance level: 0 <*** < 0.001 <** < 0.01 <* < 0.05. For the synthetic datasets (in the bottom row), the relative abundance is known from experimental design. We present this result as ‘T’. For MetaHIT, we present the performance for both Jaccard and log metric, as the latter performs much better compared with the former

*Retrieval of samples with similar annotation:* We applied the proposed approach and a number of alternatives for retrieval of similar samples from the same dataset and evaluated by how many of the retrieved samples had the same annotation: class label, disease state or body site. A comparison of the obtained MAP values averaged over queries by all positive samples is shown in Figure 4. The results show the performance achieved by the ‘optimized metric’. The alternatives we considered were—(i) retrieval performance based on the proposed distances between frequencies of 21-mers appearing in known protein families (FIGfams) with added pseudocounts but without entropy filtering (Meyer *et al.*, 2009); (ii) retrieval based on Bray–Curtis dissimilarity between relative species abundances estimated using MetaPhlAn (Segata *et al.*, 2012) (each feature is a species found in at least one samples; no regularization added); and (iii) retrieval based on $d_{S}^{2}|M_{0}$ distances between relative frequencies of 3-mers (Jiang *et al.*, 2012).

For the simulated data, the two classes differ only by the relative species abundance; thus, retrieval based on ground truth abundance can be considered to give an upper limit for the performance. For HIGH-C and HIGH-VAR, the proposed method performs closer to the ground truth performance than any other methods, although the difference from ground truth performance is still statistically significant. For LOW-C, the performance of all methods, except the protein family based comparison, drop compared with HIGH-C, whereas for MIXED-C the performance is again close to HIGH-C despite the presence of low coverage samples. This is an encouraging observation showing the robustness of the proposed approach to varying sequencing depths.

For the real datasets, the proposed approach yielded statistically significantly higher MAP than any of the alternatives (P<0.05) for all the datasets, except T2D-P2 where protein family based comparison works equally well. Interestingly, the abundance-based retrieval performs relatively poorly here, suggesting that the differences between the classes cannot be easily captured by species composition alone, while the proposed *k*-mer features can provide a better separation. Retrieval based on the known protein family performed fairly well, but slightly worse than the proposed approach on MetaHIT. We observe that for MetaHIT, Jaccard metric performs poorly; however, a change of metric to log significantly improves the performance for all methods. Otherwise, all metrics usually work equally well over different datasets.

**Fig. 5.**
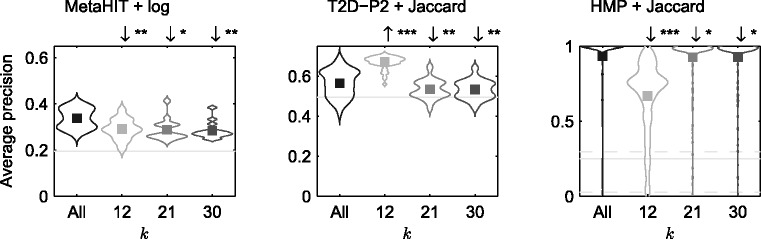
Comparison of best performances for different *k*-mer lengths. The figures show the performance over queries by all positive samples as a violin plot. All methods use the ‘optimized metric’ chosen over 101 equally spaced threshold values between 0 and 1: the box denotes the MAP value. The horizontal lines show retrieval by chance: AveP computed over zero dissimilarity metric. Straight line is the mean, and dotted lines are 5 and 95% quantiles, respectively, when number of relevant samples differ for different queries. An arrow (if present) over a method implies whether the corresponding method performs significantly better (↑) or worse (↓) than ‘All’: The stars denote significance level: 0 <*** < 0.001 <** < 0.01 <* < 0.05. We observe that the considering all *k*-mers usually perform equally well with respect to considering a single *k*

*Effect of using specific or unspecific k-*mer *length:* We next compared the proposed approach of using all *k*-mers to using a specific *k*. The retrieval performance using ‘optimized metric’ is shown in Figure 5 (and Supplementary Fig. S2). The figures show the complete distribution of average precision values over different queries whose mean is the MAP of Figure 4. The performance of the proposed method is usually better than with any individual *k*. Thus, the proposed method appears to be a relatively safe choice that does not suffer from catastrophically bad performance on any of the datasets.

**Fig. 6.**
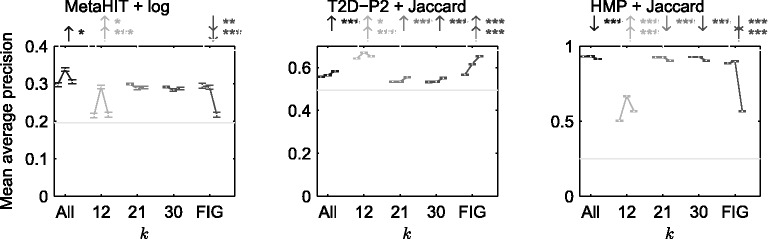
Comparison of the best retrieval performance achieved with ‘optimized metric’ (middle), ‘average metric’ (right) and without entropy filtering (left), for proposed approach ‘All’, individual *k*s as well as FIGfam-based distance metric. The metrics are ‘optimized’/‘averaged’ over 101 equally spaced threshold values between 0 and 1. Each error bar line shows the MAP value along with the standard error. The grey horizontal line shows retrieval by chance: MAP computed over zero dissimilarity metric. An arrow (if present) over a method implies whether the performance of the corresponding method (top: ‘average metric’, bottom: ‘optimized metric’) is better (↑) or worse (↓) than when entropy filtering is employed: The stars denote significance level: 0 <*** < 0.001 <** < 0.01 <* < 0.05. We observe that filtering has a positive impact on the retrieval performance

*Effect of the entropy filtering*: Next, we evaluated the efficacy of filtering the informative *k*-mers against retrieval performance without the filtering operation. The results are presented in Figure 6 (and Supplementary Fig. S3). We observed that entropy filtering usually improved retrieval performance for all tested *k*-mer lengths when using the ‘optimized metric’, although the improvement might not always be statistically significant. Although ‘average metric’ often provides significant performance, it might not always improve over performance without filtering. Also, retrieval performance of FIGfam may or may not improve with entropy filtering (‘optimized metric’ and ‘average metric’ selected in the same way as other methods).

**Fig. 7.**
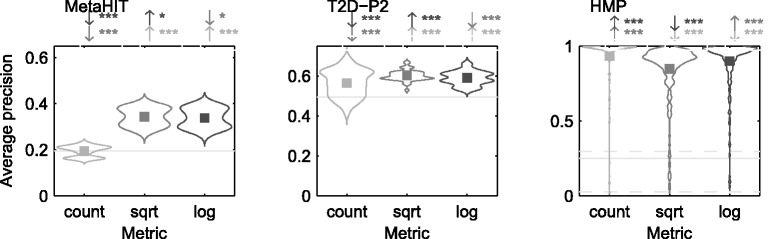
Comparison of the best retrieval performance for different distance metrics using all *k*-mers. They show a violin plot of the average performances over queries by all positive samples in the datasets. The ‘optimized metrics’ have been selected over 101 equally spaced threshold values between 0 and 1: the box denotes the MAP value. The horizontal lines show retrieval by chance: AveP computed over zero dissimilarity metric. Straight line is the mean, and dotted lines are 5 and 95% quantiles, respectively, when number of relevant samples differ for different queries. An arrow (if present) over a method implies whether the corresponding method performs significantly better (↑) or worse (↓) than the other methods (denoted by their colors): The stars denote significance level: 0 <*** < 0.001 <** < 0.01 <* < 0.05. We observe that different distance metrics usually demonstrate similar performance

*Comparison across different metrics:* Finally, we evaluated the retrieval performance over different dissimilarity metrics. We presented the performance using ‘optimized metric’ for different metrics in Figure 7 (and Supplementary Fig. S4). We observed that the simple presence-/absence-based metric *D*_count_ performed at least as well as abundance-sensitive log and sqrt metrics, except for the MetaHIT data for which the other metrics performed better.

## 5 CONCLUSION

In the wake of collecting multiple samples from similar environments, information retrieval for metagenomic samples is expected to become a handy tool in metagenomics research. In this article, we have addressed the problem of retrieving relevant metagenomic samples given a query sample from the same collection. The novelty of the proposed approach is that it is unsupervised, and does not rely on the availability of reference databases. We have suggested employing *k*-mer frequencies as feature representation; however, rather than exploring *k*-mers of a fixed *k*, we have scanned through all possible *k*-mers of all possible *k*’s using DSM, and have proposed appropriate filtering technique to discard uninformative *k*-mers. Being reference-free, our method is capable of focusing on novel sequence markers, such as ones identifying novel bacterial strains. On the other hand, sensitivity especially on low-coverage species may be lower than using reference-based approaches. Like most read-counting-based approaches, our method can be sensitive to technical variation and changing biases in the sequencing process. For most reliable results, the dataset should be measured as uniformly as possible. The distributed algorithm relies on dedicated cluster hardware having sufficient total amount of main memory. The development of an efficient secondary memory-based algorithm is an important topic of future work. The overall computational requirements scale in near-linear manner as described by Välimäki and Puglisi (2012). We have evaluated our method on both real and simulated data, and observed that the approach can effectively retrieve relevant metagenomic samples, outperforming both the FIGfams method based on known highly informative protein families as well as retrieval based on species composition of the samples.

## Supplementary Material

Supplementary Data
