# Supplementary material for: Exploration and retrieval of whole-metagenome sequencing samples
Source: Bioinformatics. 2014 May 19;30(17):2471–9. doi: 10.1093/bioinformatics/btu340 (PMC4230234; doi:10.1093/bioinformatics/btu340)
Supplement: Supplementary Data [file supp_30_17_2471__index.html]

Exploration and retrieval of whole-metagenome sequencing samples — Exploration and retrieval of whole-metagenome sequencing samples — Exploration and retrieval of whole-metagenome sequencing samples — Supplementary Data 

# Exploration and retrieval of whole-metagenome sequencing samples

## Supplementary Data

files

**Files in this Data Supplement:**

- Supplementary Data - pdf file
