# Supplementary material for: Exploration and retrieval of whole-metagenome sequencing samples
Source: Bioinformatics. 2014 May 19;30(17):2471–9. doi: 10.1093/bioinformatics/btu340 (PMC4230234; doi:10.1093/bioinformatics/btu340)
Supplement: Supplementary Data [file supp_btu340_draft-supplement.pdf]

Supplement of “Exploration and retrieval of whole-metagenome sequencing sample”

Sohan Seth [1], Niko Välimäki [2,3], Samuel Kaski [1,3] and Antti Honkela [3]
[1]Helsinki Institute for Information Technology HIIT, Department of Information and Computer Science, Aalto University, Espoo, Finland
[2]Genome-Scale Biology Program and Department of Medical Genetics, University of Helsinki, Helsinki, Finland
[3] Helsinki Institute for Information Technology HIIT, Department of Computer Science, University of Helsinki, Helsinki, Finland

site	# of samples
attached keratinized gingiva	5
buccal mucosa	27
left retroauricular crease	4
palatine tonsils	3
right retroauricular crease	13
stool	130
subgingival plaque	5
supragingival plaque	116
throat	4
tongue dorsum	128

Table 1. Number of metagenomic samples per body sites in human microbiome project (HMP) dataset used in our experiment, after removing small samples.

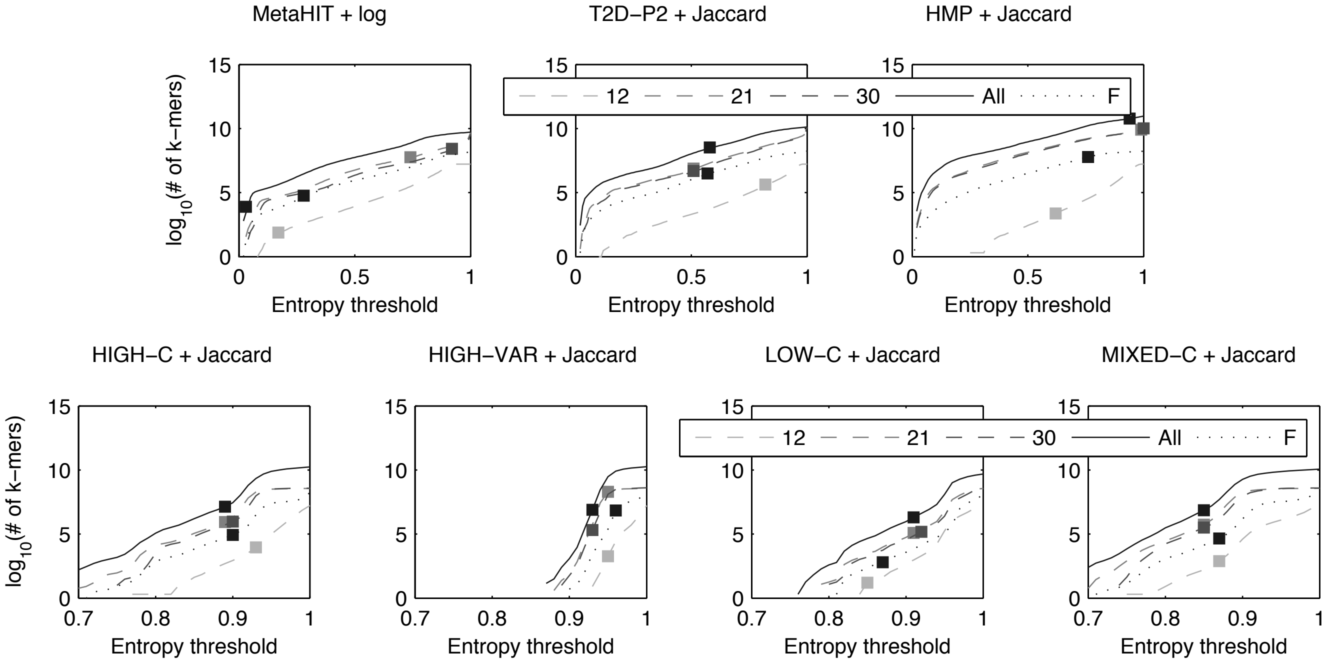

Fig. 1. Number of informative strings over varying entropy thresholds for the proposed approach ‘All’, fixed k-mer lenthgs ‘12’,‘21’ and ‘30’, and for protein family based comparison with FIGfam ‘F’. The box denotes the ‘optimized’ entropy threshold that has been used to evaluate the performance of the methods. Some general observations are as follows. The number of strings for $k = 12$ is lower than the rest while the number of strings for ‘All’ is much higher than rest of the methods, and number of strings for $k = 21$ and $k = 30$ are very close. We observe that there are strings with low entropies—more in the real data sets than in the simulated data sets—which indicate the presence of discriminative features. Also, the ‘optimized’ entropy threshold varies for different methods.

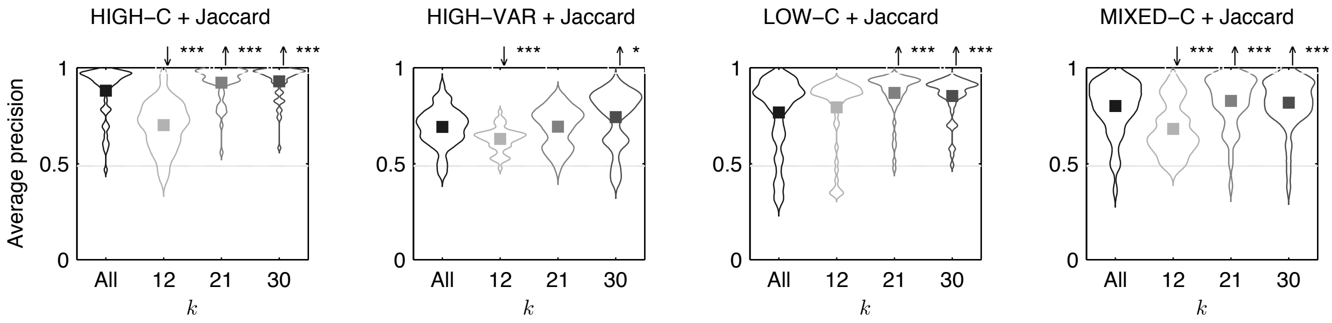

Fig. 2. Comparison of best performances for different k-mer lengths. The figures show the performance over queries by all positive samples as a violin plot. The 'optimized metrics' have been chosen in a supervised manner over 101 equally spaced threshold values between 0 and 1: the box denotes the MAP value. The horizontal lines show retrieval by chance: AveP computed over zero dissimilarity metric. Straight line is the mean, and dotted lines are 5%, and 95% quantiles respectively, when number of relevant samples differ for different queries. An arrow (if present) over a method implies whether the corresponding method performs significantly better (↑) or worse (↓) than 'All' : The stars denote significance level: $0 <$ *** $< 0.001 <$ ** $< 0.01 <$ * < 0.05. We observe that the considering all k-mers usually perform equally well with respect to considering a single k.

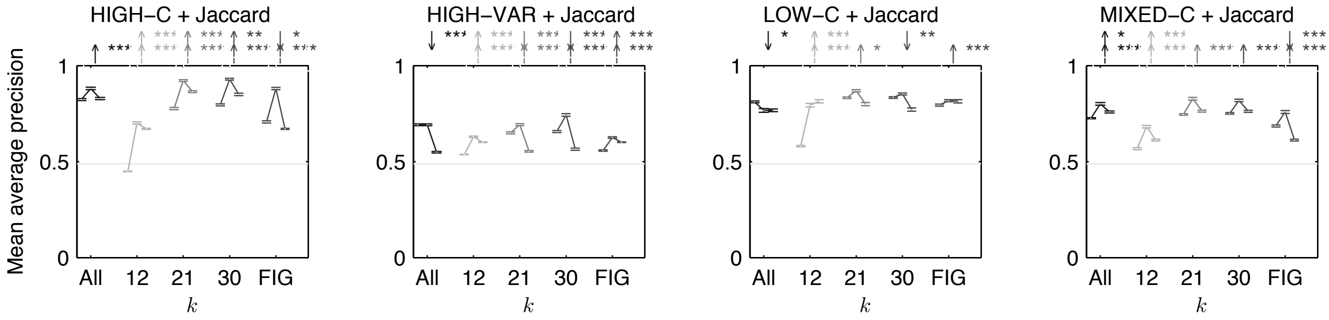

Fig. 3. Comparison of the best retrieval performance achieved with 'optimized metric' (middle), 'average metric' (right) and without entropy filtering (left), for proposed approach All, individual ks as well as FIGfam based distance metric. The metrics are 'optimized'/'averaged' over 101 equally spaced threshold values between 0 and 1. Each errorbar line shows the MAP value along with the standard error. The grey horizontal line shows retrieval by chance: MAP computed over zero similarity metric. An arrow (if present) over a method implies whether the performance of the corresponding method (top: 'average metric', bottom: 'optimized metric') improves (↑) or degrades (↓) significantly when entropy filtering is employed: The stars denote significance level: $0 <$ *** $< 0.001 <$ ** $< 0.01 <$ * < 0.05. For all cases we utilize the Jaccard metric. We observe that filtering has a positive impact on the retrieval performance.

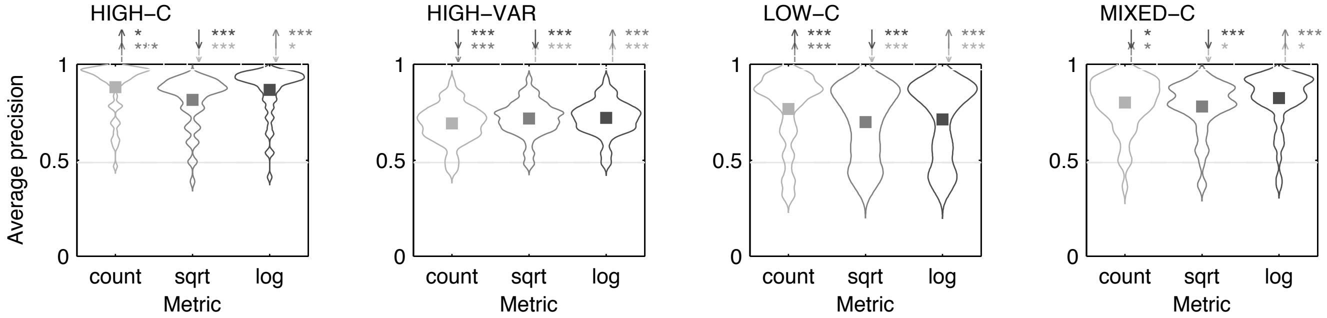

Fig. 4. Comparison of the best retrieval performance for different distance metrics using all k-mers. They show a violin plot of the average performances over queries by all positive samples in the data sets. The 'optimized metrics' have been selected over 101 equally spaced threshold values between 0 and 1: the box denotes the MAP value. The horizontal lines show retrieval by chance: AveP computed over zero dissimilarity metric. Straight line is the mean, and dotted lines are 5%, and 95% quantiles respectively, when number of relevant samples differ for different queries. An arrow (if present) over a method implies whether the corresponding method performs significantly better (↑) or worse (↓) than the other methods (denoted by their colors): The stars denote significance level: $0 <$ *** $< 0.001 <$ ** $< 0.01 <$ * < 0.05. We observe that different distance metrics usually demonstrate similar performance.
